# On Demand Urethral Dilatation Versus Intermittent Urethral Dilatation: Results and Complications in Women With Urethral Stricture

**DOI:** 10.5812/numonthly.15212

**Published:** 2014-03-10

**Authors:** Fatemeh Heidari, Shahin Abbaszadeh, Alireza Ghadian, Farahnaz Tehrani Kia

**Affiliations:** 1 Nephrology and Urology Research Center, Baqiyatallah University of Medical Sciences, Tehran, IR Iran

**Keywords:** Urethral Stricture, Urethral, Urethral, Cystoscopy

## Abstract

**Background:**

The treatment of urethral stricture in female patients is through dilatation of the urethra by tubes of increasing diameter. There are two main methods: intermittent dilatation and on demand dilatation.

**Objectives:**

The main aim of this study was to compare the results of these two methods, and to determine the best one.

**Patients and Methods:**

In this clinical trial study, we reviewed the documents of women diagnosed with urethral stricture, who came to the Baqiyatallah Clinic from 2007 and 2012. According to the method of dilatation, the patients were divided into two groups: intermittent dilatation and on demand dilatation. Patients’ data were then collected and analyzed.

**Results:**

Eighty-six patients were enrolled in the study. The mean age of the participants was 48.13 years (between 44 and 79 years). The mean urinary residual and maximum urinary flow speed changes, before and after on demand dilatation, were higher than in the intermittent method.

**Conclusions:**

For treating urethral stricture, on demand urethral dilatation is more effective than intermittent dilatation.

## 1. Background

Urethral stricture is a narrowing of the urethral lumen. It is a relatively common condition in females, but in contrast to men, it has a lower frequency. If the diameter of the urethral lumen becomes smaller than 5 mm, it is considered to be abnormal (1). There have been many studies concerning urethral stenosis in male patients that discuss different methods for the treatment of this problem. However, due to its lower prevalence in women (compared with men), there are few studies that discuss the etiology and treatment approaches of urethral stenosis in female patients. Stricture can exist in any part of the urethra or it can involve the entire urethra.

Inflammatory responses to any trauma in the urethra can induce scar formation and this results in stenosis, or even obstruction of the urethra (2). The standard treatment procedure for urethral strictures in female patients is urethral dilatation. Some surgeons perform urethral intermittent dilatation to prevent recurrent stenosis, but others prefer to repeat dilatations only after recurrences. However, there have been no studies comparing these two methods. Therefore, we conducted this study to compare on demand urethral dilatations with intermittent dilatation, for the treatment of urethral stenosis in women.

## 2. Objectives

The main aim of this study was to compare the results of these two methods and to determine the best method.

## 3. Patients and Methods

### 3.1. Patient Selection and Data Collection

In this prospective study, we operated on 86 women with primary urethral stricture, who were referred to the Baqiyatallah Hospital, between the years 2007 and 2012. A diagnosis of urethral stricture was suspected during the history taking (complaining of lower urinary tract symptoms and/or a previous history of urinary tract infection) and then patients were evaluated by ultrasonography. The criteria were: hydronephrosis, bladder mucosa thickness, and volume of post-void residue (PVR). A PVR greater than 60 mL was considered significant, and patients were also evaluated by uroflowmetry and urethrocystoscopy. In uroflowmetry, a peak flow rate (PFR, Qmax) lower than 18 mL/sec for women 14-45 years, and 15 mL/sec for women 46-80 years, were considered abnormal. A urethral dilatation was performed for all patients at the first treatment session. Thereafter, the patients were allocated randomly into two groups: on demand urethral dilatations (group 1), and intermittent urethral dilatation (group 2). In the patients of group 1, repeat urethral dilatations were performed only if symptoms relapsed. For patients in group 2, repeat urethral dilatations were performed three times, with two month intervals between each dilatation session. Exclusion criteria were; a history of previous urethral stricture treatment or incontinence treatment, history of pelvis radiotherapy, and chronic untreated gynecologic problems.

### 3.2. Surgical Technique of Urethral Dilatation

A urethrocystoscopy was performed after a general anesthesia and in the lithotomy position. The urethra was dilated with urethral dilators to 24 Fr for all patients. For patients who were allocated to the intermittent dilatation group, the procedure was repeated every two months. For patients with the on demand plan, urethral dilatation was performed after a recurrence of urethral stricture.

### 3.3. Statistics

Patients’ data included; age, post-void residual urine (PVR), maximum peak flow rate (PFR), previous history of urinary tract infection (UTI), and past medical history (normal vaginal delivery (NVD), caesarean section (C/S), and/or gynecologic surgery). All of these parameters were evaluated before and after the treatment. To analyze the data, SPSS software (version 19) was employed. Then a Mann-Whitney test was used to compare parameters between the variables of the study group. In all tests, a P value smaller than 0.05 was considered significant.

### 3.4. Follow up

Patients were re-evaluated by; history, ultrasonography, and uroflowmetry, six months after the operation.

## 4. Results

### 4.1. Demographic and Past Medical History

A total of 86 patients were included in our study, with a mean age of 48.13 ± 15.12 years (14 - 79 years). The patients’ past medical histories are shown in Table 1. Forty patients did not have any kind of gynecologic procedure. There were 47 patients (54.7%) who had a history of normal vaginal delivery (NVD), while only 2 (2.3%) patients had a history of caesarean section (C/S) without NVD. Fourteen patients had a history of both NVD and C/S, and of these; 11 patients had one C/S, 2 patients had two C/S, and 1 patient had three C/S, while 23 patients had no history of pregnancy (Table 2). 

**Table 1.** Past Medical History of Participants ^a^

	Yes		No	
	Number	Percent	Number	Percent
**Hysterectomy**	24	27.9	62	72.1
**Other gynecologic procedure**	30	34.9	56	65.1
**UTI**	47	54.7	39	45.3

^a^ Abbreviations: UTI, urinary tract infection.

**Table 2.** Delivery History of Patients ^a^

Delivery History	Number of Patients	Percent
**No delivery**	23	26.7
**Only NVD**	47	54.7
**Only C/S**	2	2.3
**NVD + C/S**		
1 C/S	11	12.8
2 C/S	2	2.3
3 C/S	1	1.2
**Total**	86	100

^a^ Abbreviations: C/S, caesarean section; NVD, normal vaginal delivery.

### 4.2. Ultrasonography, Post Void Residue

In all 86 patients, the mean PVR before and after treatment were; 92.07 mL and 44.88 mL, respectively, and this showed a statistically significant difference (P value: 0.000). For group 1, the difference of PVR before and after treatment was 48.95 mL, while for group 2 it was 45.42 mL. After analyzing with a Mann-Whitney test, the difference was found to be statistically significant (P value: 0.049).

### 4.3. Uroflowmetry, Peak Flow Rate

Mean peak flow rate (PFR) of all patients before treatment was 8.2 mL/sec, which increased to 12.72 mL after treatment, and this increment was statistically significant (P value: 0.000). This increase was 5.35 mL in group 1 patients and 3.70 mL in group 2, which is statistically significant (P value: 0.001).

## 5. Discussion

To our knowledge, this study is the first study that compares on demand versus intermittent urethral dilatation in female patients with urethral strictures. In 2012, Lee et al. reported that obstetric and gynecologic operations were strong risk factors for urologic complications, including urethral stricture (3). Interestingly, in our study, the majority of patients had a previous history of NVD (70.9%), and only 2.3% had a history of C/S without NVD. It seems that pressure on the vaginal wall, during a vaginal delivery, has a significant impact on this injury and a prolonged second phase might be of greater importance. On the other hand, 40 patients (46.5%) did not have any history of gynecologic procedures. Perhaps catheterization during gynecologic operations and the resulting physical trauma to urethral mucosa is an important etiologic factor. Other studies have revealed similar findings (3-6). Regardless of their preferred method, almost all surgeons believe that the first treatment for female urethral stricture is urethral dilatation (7, 8). In a review article in 2011, the authors stated that mechanical urethral dilatation was an effective treatment for urethral stenosis in women, but recurrence would be high (1). In our work, we did not have any recurrence after six months; however, after a longer follow-up period some patients may show symptoms of recurrence. Santucci et al. performed urethral dilatation for 1,000 female patients and they reported that it was a very effective procedure and 929 patients were cured (9). We agreed with their findings, but our goal was to compare these two methods for urethral dilatation. In other studies, only the patients’ symptoms were evaluated and none had performed or analyzed ultrasonographic or uroflowmetric measurements. However, we used these paraclinical measurements to confirm improvements after treatment. Although intermittent urethral dilatation seems to be more effective than on demand dilatation, our results show that this is not true, as both PVR and PFR will experience greater improvements than in patients with on demand schedules. This might be due to a greater risk of urethral injury with frequent urethral manipulation in patients with intermittent dilatation. On the other hand, clinical and paraclinical improvements after treatment in the on demand group are more significant that is due to problem relevant. However, after the first episode of treatment in the intermittent group, we performed three more dilatations regardless of whether the patients complained of problems. This might be unnecessary and considered as overtreatment, therefore, the results were less significant.

On demand urethral dilatation and intermittent urethral dilatation, are both very effective and safe treatments of female urethral stricture, but it seems that the on demand method is more effective than the other procedure. However, more studies with greater sample sizes should be conducted in the future, in order to produce a more precise conclusion.
